# The Improvement of Child and Adolescent Psychiatric Service Based On Armenian-French Experience

**DOI:** 10.1192/j.eurpsy.2026.11117

**Published:** 2026-07-31

**Authors:** K. Tataryan

**Affiliations:** YSMU, Yerevan, Armenia

## Abstract

**Introduction:**

The development of the children’s mental health service has received a new breath in Armenia during last years. There was not enough developed child and adolescent psychiatric service in Armenia till 2021 when in October the first inpatient psychiatric department for children was opened at Avan Mental Health Center in Yerevan.

**Objectives:**

The best motivation for young specialists to boost was the agreement between Yerevan State Medical University and East Paris Creteil University initiated by action Santa Armenie signed on 06.08.2021. It supposed to provide an exchange of French experience for several Armenian specialists. This program which was aimed to facilitate the psychiatric service for children and adolescents gave us an opportunity to visit several mental health hospitals and institutions in France in order to organize the most appropriate and perspective exchange plan for young specialists and for Armenia.

**Methods:**

The group of mental health care providers was developed under the guidance of head of psychiatry department at YSMU.

The purpose of the group was to provide mental health service at different places as well, like at home, at scholl etc, for specific cases when there are some reasons for that or when teenagers refuse to go to specialists. Each specialist has their own task and they work not only with teenager but also with family when it is necessary. Thus, the model of mobile team was one of our priorities. The other problem in Armenia among adolescents is day by day increasing addiction disorders and we paid attention to center of addictology as well. Aside from professional activity, we had a chance to enjoy Paris for 2 weeks. So exchange of experience between specialists of the two countries.

**Results:**

Thus, Armenian-French cooperation has been carried out and it is extremely important for the development of the children’s psychological-psychiatric service, and the continuous nature of cooperation is very significant point and let us to thinkand organize the next exchange program at the center of addictology in the future. We can conclude that Child and adolescent psychiatry in Armenia may have modern and multidisciplinary approaches towards different mental health problems.

The trained specialists will be able to share their knowledge and experience with colleagues by organizing various trainings, meetings and will also maintain contact with French specialists. Therefore, the is very important in order to make the reforms in the mentioned field even more realistic.

**Conclusions:**

This program which was aimed to facilitate the psychiatric service for children and adolescents gave us an opportunity to visit several mental health hospitals and institutions in France in order to organize the most appropriate and perspective exchange plan for young specialists and for Armenia.

**Disclosure of Interest:**

None Declared

